# Circulating Biomarkers for Cardiovascular Disease Risk Prediction in Patients With Cardiovascular Disease

**DOI:** 10.3389/fcvm.2021.713191

**Published:** 2021-10-01

**Authors:** Yuen-Kwun Wong, Hung-Fat Tse

**Affiliations:** ^1^Department of Medicine, The University of Hong Kong, Queen Mary Hospital, Hong Kong, China; ^2^Department of Medicine, Shenzhen Hong Kong University Hospital, Shenzhen, China; ^3^Hong Kong-Guangdong Joint Laboratory on Stem Cell and Regenerative Medicine, The University of Hong Kong, Hong Kong, China; ^4^Shenzhen Institutes of Research and Innovation, The University of Hong Kong, Hong Kong, China

**Keywords:** adipocyte, B-type natriuretic peptide, cardiac troponin, coronary artery disease, fibroblast growth factor, lipocalin, plasminogen activator inhibitor, risk prediction

## Abstract

Cardiovascular disease (CVD) is the leading cause of death globally. Risk assessment is crucial for identifying at-risk individuals who require immediate attention as well as to guide the intensity of medical therapy to reduce subsequent risk of CVD. In the past decade, many risk prediction models have been proposed to estimate the risk of developing CVD. However, in patients with a history of CVD, the current models that based on traditional risk factors provide limited power in predicting recurrent cardiovascular events. Several biomarkers from different pathophysiological pathways have been identified to predict cardiovascular events, and the incorporation of biomarkers into risk assessment may contribute to enhance risk stratification in secondary prevention. This review focuses on biomarkers related to cardiovascular and metabolic diseases, including B-type natriuretic peptide, high-sensitivity cardiac troponin I, adiponectin, adipocyte fatty acid-binding protein, heart-type fatty acid-binding protein, lipocalin-2, fibroblast growth factor 19 and 21, retinol-binding protein 4, plasminogen activator inhibitor-1, 25-hydroxyvitamin D, and proprotein convertase subtilisin/kexin type 9, and discusses the potential utility of these biomarkers in cardiovascular risk prediction among patients with CVD. Many of these biomarkers have shown promise in improving risk prediction of CVD. Further research is needed to assess the validity of biomarker and whether the strategy for incorporating biomarker into clinical practice may help to optimize decision-making and therapeutic management.

## Introduction

Individuals with stable coronary artery disease (CAD) are at higher risk of recurrent cardiovascular event and mortality than the general population. Preventive strategies and intensive management of cardiovascular risk factors are much needed to improve the prognosis of these patients. Although conventional risk prediction models such as Framingham Risk Score have been developed and widely used to estimate individual's risk for primary prevention of cardiovascular disease (CVD) (1), effective tools for risk assessment in secondary prevention are still missing. The mechanisms underlying the increased risk of recurrent CVD are not fully understood. Existing prediction models that based on traditional risk factors such as age, gender, diabetes status, blood pressure, cholesterol levels, and smoking status may have limited value to risk stratify patients with stable CAD (2).

Circulating biomarkers such as high sensitivity C-reactive protein and cardiac troponin have been playing a crucial role in the diagnosis, risk stratification, and management of patients with several disease conditions including heart failure (HF) and acute coronary syndrome (ACS) (3, 4). Recently, numerous novel biomarkers from different pathophysiological pathways have been found to be associated with cardiovascular risk and may provide important prognostic information (5–7). The combined use of multiple biomarkers has also proven to be useful in the risk stratification of CVD (8). In this review, we focus on the potential utility of various biomarkers from cardiac- and metabolic-related pathways for predicting cardiovascular risk in secondary prevention setting. The reviewed biomarkers include: (i) cardiac-related biomarkers [B-type natriuretic peptide (BNP), N-terminal pro-B-type natriuretic peptide (NT-proBNP), and cardiac troponin I (cTnI)]; and (ii) metabolic-related biomarkers [adiponectin, adipocyte fatty acid-binding protein (A-FABP), heart-type fatty acid binding protein (H-FABP), lipocalin-2, fibroblast growth factor (FGF) 19 and 21, retinol-binding protein 4 (RBP4), plasminogen activator inhibitor-1 (PAI-1), 25-hydroxyvitamin D, and proprotein convertase subtilisin/kexin type 9 (PCSK9)]. These biomarkers are of special interest as they are thought to provide sufficient information for improving cardiovascular risk stratification. Evolving biomarkers such as non-coding RNAs are beyond the scope of this review, although they have shown a potential in this field (9). The potential mechanistic link between biomarkers and CVD are summarized in Table 1.

**Table 1:** Potential mechanistic link between CVD and biomarkers.

**Biomarker**	**Potential link with CVD**	**References**
Cardiac troponin I	Myocardial injury	(10)
BNP/NT-proBNP	Myocardial stretch	(11)
Adiponectin	Insulin resistance Altered lipid metabolism Endothelial dysfunction Atherosclerosis Inflammation	(12, 13)
A-FABP	Insulin resistance Altered lipid metabolism Endothelial dysfunction Atherosclerosis Inflammation	(14, 15)
H-FABP	Altered lipid metabolism Myocardial injury	(16)
Lipocalin-2	Atherosclerosis Plaque instability Vascular remodelling Insulin resistance Inflammation	(17, 18)
FGF-19	Altered lipid metabolism Altered glucose metabolism Insulin resistance	(19, 20)
FGF-21	Altered lipid metabolism Altered glucose metabolism Insulin resistance	(19, 21)
RBP4	Insulin resistance Atherosclerosis Inflammation	(22, 23)
PAI-1	Thrombus formation Impaired fibrinolysis Insulin resistance Inflammation	(24)
25-hydroxyvitamin D	Insulin resistance Endothelial dysfunction Atherosclerosis Inflammation	(25)
PCSK9	Altered lipid metabolism Atherosclerosis	(26)

*A-FABP, adipocyte fatty acid-binding protein; BNP, B-type natriuretic peptide; FGF, fibroblast growth factor; H-FABP, heart-type fatty acid-binding protein; NT-proBNP, N-terminal pro-B-type natriuretic peptide; PAI, plasminogen activator inhibitor; PCSK9, proprotein convertase subtilisin/kexin type 9; RBP, retinol-binding protein*.

## Characteristics of Biomarker

A biomarker, or biological marker, is broadly defined as a “characteristic that is objectively measured and evaluated as an indicator of normal biological processes, pathogenic processes, or pharmacologic responses to a therapeutic intervention” (27). Biomarkers can be classified into four types: diagnostic biomarkers are expected to facilitate the early detection of disease; prognostic biomarkers are used for estimating the likely course of the disease; predictive biomarkers are used to predict patient's response to a particular therapy; therapeutic biomarkers help to identify new therapeutic targets (28). Biomarkers can also be used as a substitute for a clinical endpoint in clinical trials. The desired characteristics of biomarkers vary based on their intended use. For instance, high specificity is required if a biomarker is used for screening purpose. As stated by Morrow and de Lemos, biomarker should fulfill a set of criteria to be clinically useful: (1) it must be accurate, reproducible, easy to obtain and inexpensive; (2) it must provide added value over existing measures; (3) it must aid in clinical decision-making (29).

## Statistical Assessments for the Evaluation of Biomarker Performance

Several statistical measures have been proposed for evaluating the utility of a new biomarker. The statistical association between a biomarker and the outcome can be assessed using metrics such as odds ratio, relative risk or hazard ratio. Statistical significance of an association is necessary but insufficient to provide information regarding the clinical contribution or usefulness of a new biomarker (30). Other measures including discrimination, calibration and reclassification are recommended for assessing the incremental contribution of a new biomarker to a conventional risk prediction model.

Discrimination refers to the ability of a biomarker to distinguish individuals who develop a disease from those who do not (31). The area under the receiver operating characteristic (AUC), which is equivalent to the c statistic, is the most used measure of model discrimination (32). The AUC is the probability that a randomly chosen individual with the disease has a higher predicted risk than a randomly chosen individual without the disease. Values for AUC range from 0.5 (no discrimination) to 1.0 (perfect discrimination). In general, the AUC > 0.7 indicates a good model. The increase in AUC can also be used to quantify the added predictive value offered by the new biomarker. However, the AUC is relatively insensitive to small improvements in model performance when the AUC of the baseline model is well-discriminated (33).

Calibration is also an important measure of model accuracy. It measures the ability of the model to accurately predict the proportion of individuals in a group who will develop the disease events. A risk prediction model is well-calibrated when the predicted probabilities agree with the observed frequencies of an event. Statistical metric of Hosmer-Lemeshow χ^2^ test is commonly used for assessing the calibration of a risk prediction model (34). A *P* < 0.05 for Hosmer-Lemeshow test indicates poor calibration of the model.

Reclassification refers to the ability to reclassify individuals into different risk categories. The reclassification measures including net reclassification index (NRI) and integrated discrimination improvement (IDI) have been proposed to quantify how well a new biomarker improves risk classification and as alternatives to the AUC (35). NRI is the net proportion of individuals with the event correctly reclassified “upward” (i.e., moving up to higher risk category) and the net proportion of individuals without the event correctly reclassified “downward” (i.e., moving down to lower risk category). This category-based NRI is highly sensitive to the number of risk categories and the choice of risk thresholds. Pencina et al., therefore, proposed a category-free version of the NRI to overcome the problem of selecting categories (36). Positive values of NRI indicate improved reclassification and negative values indicate worsened reclassification. On the other hand, IDI is independent of risk category and defined as the difference in discrimination slopes between models with and without the new marker (35). Discrimination slope is calculated as the difference between the average predicted probabilities for events and non-events.

In summary, there is no single statistical method can be used for evaluating the incremental value of a new biomarker. The metrics that used should be depending on the needs and objectives.

## Methods

### Search Strategy

A literature search was conducted using PubMed to identify all relevant studies. Research articles were also selected manually from the reference lists of articles. The search strategy used the terms “biomarker,” “coronary artery disease,” “cardiovascular disease,” “metabolic disease,” “cardiac troponin,” “natriuretic peptide,” “heart-type fatty acid-binding protein,” “adipokines,” “adiponectin,” “fibroblast growth factor,” “fatty acid binding protein,” “lipocalin 2,” “neutrophil gelatinase-associated lipocalin,” “retinol binding protein,” “plasminogen activator inhibitor,” “vitamin D,” “PCSK9,” and “risk prediction” in several combinations. Duplicated studies were identified and removed using Endnote duplicate function. The abstracts and titles of article retrieved were screened to exclude the irrelevant studies. Full-text articles were then examined to determine whether they met the inclusion criteria.

### Inclusion and Exclusion Criteria

Inclusion criteria were: (1) studies investigating the association of a biomarker with metabolic and cardiovascular diseases, and adverse clinical outcomes such as cardiovascular events and death; (2) studies using blood serum or plasma for biomarker analysis; and (3) peer-reviewed articles and all types of reviews published in English between January 1980 and November 2020. Unpublished theses, reports, and conference proceedings were excluded. Animal studies were also excluded.

### Data Extraction and Quality Assessment

Due to the heterogeneity of focus and results from the refined studies, we did not perform a meta-analysis as part of the review process. Data were extracted using a standardized form by one reviewer and verified by a second reviewer. The following data were extracted from eligible studies: first author, year of publication, country, study design, population characteristics and sample size, specimen type, follow-up duration, and main findings. The Newcastle-Ottawa Scale was used to assess the quality of the selected cohort and case-control studies, with a maximum score of nine points (37). The quality of the cross-sectional studies was assessed using the adapted version of the Newcastle-Ottawa Scale that awards a maximum score of 10 points (38). The Newcastle-Ottawa Scale assesses three main domains: selection, comparability, and outcome assessment. The AMSTAR-2 (A Measurement Tool to Assess Systematic Reviews-2) was used to evaluate the methodological quality of systematic reviews (39).

## Results

### Study Identification

**Figure 1:**
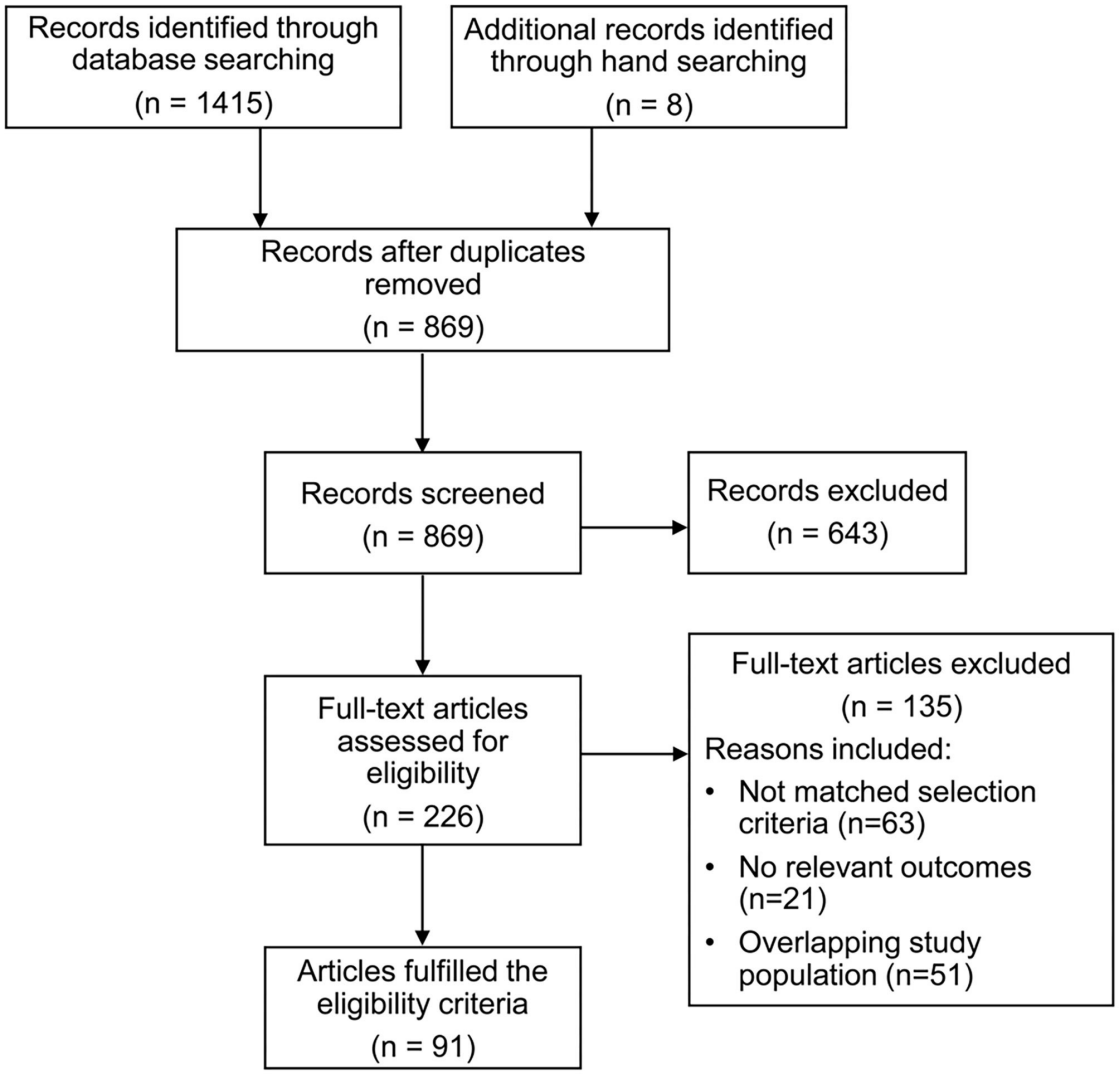
Flowchart of study selection process.

The study selection process is summarized in Figure 1. A total of 1,423 records were identified through the initial literature search. After removing 554 duplicates, the remaining 869 articles were screened, and 643 articles were excluded. The remaining 226 full-text articles were retrieved for detailed assessment. Ninety-one articles were identified to fulfill the eligibility criteria and were included in the final analysis.

### Study Characteristics

The 91 included studies were conducted in 24 countries and were published from 1986 to 2020. There were 43 cohort studies, 36 cross-sectional studies, 7 case-control studies, and 5 meta-analyses. The sample size of these observational studies ranged from 22 to 41,504, with a total of 135,811 participants. The following biomarkers were studies: 6 studies investigated cardiac troponin I, 10 investigated BNP or NT-proBNP, 9 reported on adiponectin, 8 reported on A-FABP, 5 reported on H-FABP, 12 on lipocalin-2, 12 on FGF-19 and/or FGF-21, 7 assessed RBP4, 7 assessed PAI-1, 8 reported on vitamin D, and 7 on PCSK9.

### Quality Assessment

The results of study quality assessment are presented in Supplementary Table 1 for the cohort studies, in Supplementary Table 2 for the case-control studies, in Supplementary Table 3 for the cross-sectional studies, and in Supplementary Table 4 for the meta-analyses. According to the Newcastle-Ottawa Scale, 84 studies scored 7 or more points (good quality) and 2 studies scored 6 points (fair quality). Of the five included reviews, according to the AMSTAR-2 rating, three were rated as moderate or high quality and two were rated as low or critically low quality.

## Role of Biomarkers in Cardiovascular Risk Assessment

### Cardiac Troponin I

cTnI is one of the subunits of troponin regulatory complex that exclusively expressed in cardiac muscle, and released into the bloodstream after cardiac injury. cTnI is an established biomarker and clinically used as gold standard for the detection of myocardial injury (10). Increased levels of cTnI can be found in a variety of cardiac and non-cardiac conditions, including myocardial infarction, HF, pulmonary embolism, myocarditis, sepsis, and renal failure (40). Several studies have demonstrated that elevated high-sensitivity cardiac troponin I (hs-cTnI) levels in patients with HF were associated with poor prognosis and increased risk of mortality (41, 42). The addition of hs-cTnI to a traditional risk factor model improved the AUC by 0.05 for subsequent HF and cardiac death (43). Moreover, levels of hs-cTnI independently predicted adverse cardiovascular events in type 2 diabetes mellitus (T2DM) patients with ACS. Patients with hs-cTnI levels >99th percentile demonstrated a 4-fold higher risk of major cardiovascular events (44). Among patients with stable CAD, hs-cTnI has been shown to predict subsequent myocardial infarction and cardiovascular death during a median follow-up of 6 years (45). In a prospective study of patients with CAD, elevated hs-cTnI levels were higher in patients with more severe CAD, and were independently associated with adverse cardiovascular events and mortality. Addition of hs-cTnI improved the AUC by 0.03 and an NRI of 25% (46). These findings showed that hs-cTnI levels had an additive prognostic value for future cardiovascular outcomes over a conventional model with clinical risk factors. Supplementary Table 5 summarizes the studies on the predictive value of cTnI.

### B-Type Natriuretic Peptide

BNP is a protein secreted by the cardiac ventricles in response to increased ventricular stretch or wall stress. It is also involved in regulating volume homeostasis and cardiovascular remodeling (47). BNP is synthesized as proBNP and is cleaved into active BNP and more stable NT-proBNP within cardiomyocytes. NT-proBNP has a longer half-life and lower variation than BNP. The clinical utility of BNP and NT-proBNP is largely similar (11). BNP and NT-proBNP are widely used for the diagnosis and risk stratification in patients with HF (48). Circulating BNP levels are lower in obese than in non-obese patients, and inversely correlated with body mass index (49). Higher levels of BNP have been found in patients with left ventricular hypertrophy and myocardial infarction (50). It has been proven that BNP level provides important prognostic information in patients with CAD, T2DM, and hypertension (51–53). Among patients with ACS and T2DM, BNP has been shown to be a powerful predictor of cardiovascular death, regardless of prior history or HF or any prior CVD (54). Another study has demonstrated that HF patients with elevated levels of BNP and cardiac troponin were at particularly high risk for mortality (55). Previous studies have also found that elevated BNP levels were associated with increased risk of adverse cardiovascular events and mortality in patients with CAD. The addition of BNP to a traditional risk factor model improved the AUC by 0.02 for prediction of adverse cardiovascular events (51, 56). Multi-marker approach based on NT-proBNP and cardiac troponin was associated with adverse events after adjustment for cardiovascular risk factors. The model incorporating a combination of NT-proBNP and cardiac troponin resulted in increases in the AUC, NRI, and IDI, suggesting that these biomarkers may serve as independent prognostic markers for CVD risk prediction (57). Supplementary Table 6 summarizes the studies on the predictive value of BNP/NT-proBNP.

### Adiponectin

Adiponectin is an adipokine secreted by adipose tissues and exhibits anti-inflammatory, anti-atherogenic, and cardioprotective effects (12, 13). Adiponectin expression is reduced in obesity, insulin resistance, and T2DM, and the plasma level is inversely related to body mass index and components of metabolic syndrome such as triglycerides and insulin levels (58, 59). Lower adiponectin levels are associated with endothelial dysfunction, increased carotid intima-media thickness (IMT) and severity of CAD (60–62). Several studies have demonstrated that adiponectin could serve as a risk factor for CVD and had moderate accuracy for the identification of metabolic syndrome, with AUC ranged from 0.67 to 0.89 (63). Circulating adiponectin has also been shown to predict cardiovascular and all-cause mortality risk in patients with prevalent CVD (64). In patients with ACS, adiponectin was associated with higher risk of adverse cardiovascular outcomes (65). Another prospective study of patients with stable CAD also reported that higher level of adiponectin was associated with a 6-fold increased risk of all-cause mortality, with good discrimination ability (AUC, 0.78) (66). Supplementary Table 7 summarizes the studies on the predictive value of adiponectin.

### Adipocyte Fatty Acid-Binding Protein

A-FABP is mainly expressed in adipocytes and macrophages, and has an important role in regulating glucose and lipid metabolism (14). Circulating A-FABP levels are closely linked to the development of obesity, insulin resistance, diabetes, hypertension, cardiac dysfunction, and atherosclerosis (15, 67). Elevated A-FABP levels are found in patients with CAD, and are positively correlated with metabolic syndrome and severity of coronary atherosclerosis (68, 69). Recent studies have shown that increased A-FABP concentrations were independently associated with increased risk of adverse cardiovascular events and cardiovascular mortality in patients with CAD (70–72). The association between A-FABP levels and cardiovascular events has also been observed in a prospective study with median follow-up of 9.4 years (73). Subjects with elevated A-FABP levels showed a 1.6-fold increased risk of cardiovascular events. The NRI and IDI were significantly improved by adding A-FABP to a traditional risk factor model (NRI, 18.6%; IDI, 0.25%). In another prospective study of patients with ACS, A-FABP was associated with a higher risk of adverse events, and demonstrated that the model with a combination of A-FABP and NT-proBNP may provide a better predictive performance than A-FABP alone, with the AUC increased from 0.65 to 0.68 (74). Supplementary Table 8 summarizes the studies on the predictive value of A-FABP.

### Heart-Type Fatty Acid-Binding Protein

H-FABP is a low molecular-weight cytoplasmic protein that is abundant in the myocardium. H-FABP is released rapidly into the circulation in response to myocardial injury, and is therefore used as an early and sensitive diagnostic marker for myocardial infarction (16). It has been reported that serum H-FABP levels are elevated in patients with metabolic syndrome and pre-diabetic patients, and positively correlated with carotid IMT (75, 76). Circulating H-FABP level has also been shown to be a strong predictor of major cardiac events and mortality in patients with ACS, suggesting that H-FABP may provide incremental information for cardiovascular risk stratification that was independent of traditional risk factors, troponin I, and BNP (77). In patients with chronic heart failure, high H-FABP was associated with 5.4-fold higher risk cardiac events, and had a higher predictive value than BNP (AUC, 0.79 vs. 0.67) (78). A recent prospective study comprising of 4,594 patients with stable CAD showed that high levels of H-FABP were associated with increased risk of adverse cardiovascular events, and found a greater risk in CAD patients with impaired glucose metabolism (79). Supplementary Table 9 summarizes the studies on the predictive value of H-FABP.

### Lipocalin-2

Lipocalin-2, also known as neutrophil gelatinase-associated lipocalin, belongs to the lipocalin superfamily, and was first identified in the specific granules of neutrophils (80). Lipocalin-2 is expressed in a various tissues including liver, kidney, lung, adipose tissue, stomach, and small intestine (81). There is also evidence to suggest that lipocalin-2 may play a role in vascular remodeling and plaque instability in atherosclerosis (17). Circulating lipocalin-2 levels are elevated in obese patients and patients with T2DM, and positively correlated with insulin resistance index and inflammatory markers (18, 82, 83). It has been reported that high levels of lipocalin-2 are associated with markers of atherosclerosis, presence and severity of CAD (84–86). In a population-based cohort study, lipocalin-2 level was an independent predictor of cardiovascular events in male subjects. The addition of lipocalin-2 to traditional risk factors improved the AUC from 0.77 to 0.81 (87). Serum lipocalin-2 levels were higher in patients with CAD or chronic HF compared with the healthy individuals (88, 89). Several studies have reported that elevated lipocalin-2 level was associated with increased risk of cardiovascular and all-cause mortality in patients with ST-segment elevation myocardial infarction after adjustment for conventional risk factors, with AUC ranging from 0.76 to 0.85, indicating a good predictive ability for prediction of mortality in these patients (90, 91). Elevated level of lipocalin-2 has also been found to be associated with a 4-fold higher risk of mortality in a 2-year follow-up study of patients with HF (92). Supplementary Table 10 summarizes the studies on the predictive value of lipocalin-2.

### Fibroblast Growth Factor 19 and 21

FGF-19 and FGF-21 belong to the same subfamily of endocrine FGFs. The FGF family comprises of 22 members, which are classified into seven subfamilies based on the structural characteristics and mechanisms of action (93). FGF-19 is primarily secreted by the small intestine during feeding, and FGF-21 is secreted by the liver during fasting, with both FGF-19 and FGF-21 share similar functions in regulating lipid, glucose and energy metabolism (19). It has been shown that circulating levels of FGF-19 are decreased in obese patients and T2DM patients with metabolic syndrome, and are inversely correlated with fasting glucose levels (20, 21, 94). In a study of 315 patients, serum FGF-19 levels were significantly lower in patients with CAD than those in the control group, and were independently associated with severity of CAD (95). On the other hand, levels of FGF-21 are elevated in patients with T2DM and those with established CAD, and are strongly associated with body mass index, triglycerides, insulin resistance, and serum A-FABP levels (96, 97). High FGF-21 level has also been reported to be an independent predictor of the development of T2DM and metabolic syndrome (98, 99). A prior study recruited individuals who underwent carotid IMT assessment demonstrated that elevated FGF-21 levels were associated with the presence of carotid atherosclerosis (100). Serum FGF-21 level was increased in patients with acute myocardial infarction compared to the control group, and associated with a higher risk of adverse cardiovascular event after follow-up of 24 months. The predictive performance of FGF-21 level was modest with an AUC of 0.67 (101). In patients with CAD, elevated FGF-21 level was associated with increased risk of cardiovascular events and mortality after adjustment for traditional cardiovascular risk factors (102, 103). Supplementary Table 11 summarizes the studies on the predictive value of FGF-19 and FGF-21.

### Retinol-Binding Protein 4

RBP4 is a member of the lipocalin family and the sole retinol transporter in blood. It is mainly secreted by the human liver and adipose tissue (104). Previous studies have revealed that RBP4 concentrations were elevated in patients with obesity and T2DM, and were associated with insulin resistance (105). Other studies have also demonstrated strong correlations of increased RBP4 levels with carotid IMT and components of the metabolic syndrome including hypertension, hypertriglyceridemia, and waist circumference, suggesting that RBP4 may serve as a marker of metabolic complications and atherosclerosis (22, 23, 106). Moreover, circulating RBP4 levels have been shown to be correlated with CVD. A recent study reported that RBP4 levels were higher in patients with CAD than those in control subjects, and were positively correlated with the prevalent and severity of CAD (107). Elevated RBP4 level was associated with an increased risk of CAD in a 16-year follow-up study of women subjects (108). It has also been reported that serum RBP4 level is an independent predictor of adverse cardiovascular events in patients with chronic HF after adjustment for cardiovascular risk factors, and shows good prognostic performance with an AUC of 0.74 (109). Supplementary Table 12 summarizes the studies on the predictive value of RBP4.

### Plasminogen Activator Inhibitor-1

PAI-1, a member of the serine protease inhibitor (serpin) family, is the primary inhibitor of both the tissue-type and the urinary-type plasminogen activator (110). PAI-1 is mainly secreted by endothelial cells and various tissue types such as liver and adipose tissue. It is also involved in various physiological and pathological processes including fibrinolysis, tissue modeling, cancer, inflammation and CVD (24, 111, 112). Circulating levels of PAI-1 are increased in obesity, insulin resistance, and T2DM (113, 114). Elevated plasma PAI-1 levels have been reported to be an independent predictor of CVD in patients with myocardial infarction (115). Recently, a study revealed that elevated PAI-1 level was causally associated with incident CAD, suggesting that PAI-1 may have a role in the pathogenesis of CAD (116). Several studies have also demonstrated that elevated PAI-1 levels were associated with adverse cardiovascular events in patients with established CAD (117). In a prospective study of patients with ST-elevation myocardial infarction, high PAI-1 level was associated with a 5.5-fold increased risk of 5-year mortality, with an AUC of 0.75 (118). Furthermore, in the study of the Framingham Offspring study, Tofler et al. showed that both baseline and serial changes in PAI-1 levels were associated with subsequent risk of CVD, but only modest improvement in the AUCs were observed when adding PAI-1 to the traditional risk factor model (119). Supplementary Table 13 summarizes the studies on the predictive value of PAI-1.

### Vitamin D

Vitamin D is a secosteroid hormone that involves in maintaining calcium and phosphorus homeostasis, and promoting bone mineralization. 25-hydroxyvitamin D concentrations is the best indicator of vitamin D status (120). Vitamin D deficiency is often associated with bone disorders such as rickets and osteoporosis. Vitamin D has also been linked to non-skeletal diseases, including cancer, CVDs, obesity, diabetes and hypertension (25). Low vitamin D level has been found to be independently associated with increased carotid IMT and presence of carotid plaque, suggesting a potential role of vitamin D in the development of atherosclerosis (121). In addition, vitamin D deficiency was found to be associated with the prevalence and severity of CAD (122). Several studies have demonstrated that low vitamin D level was associated with increased risk of cardiovascular events including myocardial infarction (123–125). In a prospective study of 41,504 individuals, vitamin D deficiency was associated with higher prevalence of diabetes, hypertension, hyperlipidemia, and peripheral vascular disease. Patients with vitamin D level below 15 ng/mL demonstrated a 2-fold higher risk of adverse outcomes than those with normal level (126). Another large prospective study also reported that low vitamin D levels were associated with increased risk of ischemic heart disease, myocardial infarction and early death (127). More recently, a study showed that serum vitamin D levels on admission were associated with in-hospital mortality in patients with acute pulmonary embolism. A cut-off level of vitamin D ≤6.47 ng/mL was optimum for the prediction of in-hospital mortality with an AUC of 0.81, suggesting that vitamin D may be a potential prognostic biomarker for pulmonary embolism (128). Supplementary Table 14 summarizes the studies on the predictive value of 25-hydroxyvitamin D.

### Proprotein Convertase Subtilisin/Kexin Type 9

PCSK9, a member of the proprotein convertase family, is predominantly produced in the liver and plays a key role in cholesterol homeostasis. It reduces the low-density lipoprotein intake from circulation by enhancing the degradation of hepatic low-density lipoprotein receptor (26). Circulating PCSK9 concentrations are elevated in patients with metabolic syndrome, T2DM, and obesity (129–131). In a study of 126 with hypertensive patients, serum PCSK9 was associated with carotid IMT (132). Several studies have reported that PCSK9 levels were associated with the severity of coronary stenosis in patients with ACS, after adjustment for established risk factors (133). In a prospective study of 1,225 patients with stable CAD, elevated PCSK9 levels were related to cardiovascular metabolic markers such as total cholesterol and hemoglobin A1c, and independently associated with increased risk of adverse cardiovascular events. Patients with T2DM and high PCSK9 levels demonstrated a 5-fold increased risk of adverse cardiovascular events compared with non-diabetic patients with low PCSK9 levels (134). The association of PCSK9 levels with cardiovascular events was also observed in patients with CAD on statin treatment (135). Supplementary Table 15 summarizes the studies on the predictive value of PCSK9.

## Discussion

Accurate risk stratification tools are important for clinical risk prediction and treatment strategy, particularly for individuals in higher risk groups. The selected biomarkers in this review are closely linked with CVD and have shown promise in improving the prediction of adverse cardiovascular events for primary and secondary prevention. However, validation of potential biomarkers on a larger scale remains challenging and their clinical utility in stable CAD patients is still to be determined. There is some controversy regarding which biomarker is more suitable for the prognosis of CAD. The multi-biomarker approach may help overcome some of the limitations of individual markers and improve the prognostic accuracy. It has been suggested that the strategy of combining biomarkers from different pathways is more likely to be clinically useful than biomarkers in the same pathway, and may provide greater discriminative ability than individual biomarker. For example, Hillis et al. demonstrated that the combined model of NT-proBNP and cardiac troponin provided better prognostic information with regard to the risk for future cardiovascular events than the use of a single biomarker (57). Reiser et al. also reported that the combination of NT-proBNP and A-FABP yielded a more accurate predictive value for adverse outcomes in patients with ACS (74). These findings provide new insights into the potential use of multiple biomarkers related to cardiovascular and metabolic pathways to improve strategies for secondary prevention of CVD.

Cost is also an important consideration when selecting biomarkers for risk prediction models. Some biomarkers can be expensive to measure and other practical issues such as collection, storage and handling of samples may affect the cost of a biomarker model. Moreover, the economic burden on healthcare system after implementation of biomarker prediction tools may include the costs of: (i) additional biomarker tests; (ii) detailed assessments for risk estimation; and (iii) new therapies or interventions for treating high-risk patients to reduce risk. Although the overall costs may be increased, it may be cost-effective if health outcomes are improved sufficiently. Further evaluation of the cost-effectiveness of using biomarker prediction tools is needed to inform health policy as well as to guide clinical decisions.

In conclusion, our study revealed that these biomarkers representing different pathophysiological pathways could help to improve risk stratification for CVD. Further work is warranted to identify optimal combination of biomarkers for risk stratification of secondary prevention patients. In addition, validation studies are still required to confirm the applicability of these biomarkers in CVD risk prediction.

## Author Contributions

Y-KW wrote the manuscript. H-FT reviewed the manuscript. Both authors read and approved the final version.

## Conflict of Interest

The authors declare that the research was conducted in the absence of any commercial or financial relationships that could be construed as a potential conflict of interest.

## Publisher's Note

All claims expressed in this article are solely those of the authors and do not necessarily represent those of their affiliated organizations, or those of the publisher, the editors and the reviewers. Any product that may be evaluated in this article, or claim that may be made by its manufacturer, is not guaranteed or endorsed by the publisher.

## Abbreviations

ACS: acute coronary syndrome
A-FABP: adipocyte fatty acid-binding protein
AUC: area under the curve
CAD: coronary artery disease
hs-cTnI: high-sensitivity cardiac troponin I
CVD: cardiovascular disease
BNP: B-type natriuretic peptide
FGF: fibroblast growth factor
HF: heart failure
H-FABP: heart-type fatty acid-binding protein
IDI: integrated discrimination improvement
IMT: intima-media thickness
NT-proBNP: N-terminal pro-B-type natriuretic peptide
NRI: net reclassification index
PAI: plasminogen activator inhibitor
PCSK9: proprotein convertase subtilisin/kexin type 9
RBP: retinol-binding protein
T2DM: type 2 diabetes mellitus.
