# Supplementary material for: Circulating Biomarkers for Cardiovascular Disease Risk Prediction in Patients With Cardiovascular Disease
Source: Front Cardiovasc Med. 2021 Oct 1;8:713191. doi: 10.3389/fcvm.2021.713191 (PMC8517145; doi:10.3389/fcvm.2021.713191)
Supplement: Supplementary file 1 [file Data_Sheet_1.docx]

| **Supplementary Table 1. Newcastle-Ottawa quality assessment scale for cohort studies** | | | | |
| --- | --- | --- | --- | --- |
| **Study** | **Selection** | **Comparability** | **Outcome** | **Total score** |
| You 2007 | 4 | 2 | 3 | 9 |
| Myhre 2018 | 3 | 2 | 3 | 8 |
| Stelzle 2017 | 2 | 2 | 2 | 6 |
| Cavender 2017 | 3 | 2 | 2 | 7 |
| White 2014 | 3 | 2 | 3 | 8 |
| Tahhan 2018 | 4 | 2 | 3 | 9 |
| Masson 2008 | 3 | 2 | 3 | 8 |
| Omland 2007 | 3 | 2 | 3 | 8 |
| Bhalla 2004 | 3 | 1 | 3 | 7 |
| Paget 2011 | 3 | 2 | 3 | 8 |
| Wolsk 2017 | 4 | 2 | 3 | 9 |
| Mishra 2014 | 4 | 2 | 3 | 9 |
| Hillis 2014 | 4 | 2 | 3 | 9 |
| Inoue 2007 | 3 | 2 | 3 | 8 |
| Oliveira 2013 | 3 | 2 | 2 | 7 |
| Pratesi 2016 | 3 | 2 | 2 | 7 |
| Huang 2018 | 3 | 2 | 3 | 8 |
| Takagi 2017 | 3 | 2 | 3 | 8 |
| Eynatten 2012 | 3 | 2 | 3 | 8 |
| Chow 2013 | 4 | 2 | 3 | 9 |
| Reiser 2015 | 4 | 2 | 3 | 9 |
| O'Donoghue, 2016 | 3 | 2 | 3 | 8 |
| Niizeki, 2005 | 3 | 2 | 3 | 8 |
| Zhang, 2020 | 3 | 2 | 3 | 8 |
| Wu 2014 | 4 | 2 | 3 | 9 |
| Yndestad 2009 | 3 | 2 | 3 | 8 |
| Akcay 2012 | 3 | 2 | 3 | 8 |
| Avci 2020 | 3 | 2 | 3 | 8 |
| Bolignano 2009 | 3 | 2 | 3 | 8 |
| Chen 2011 | 4 | 2 | 3 | 9 |
| Chen 2018 | 3 | 2 | 3 | 8 |
| Shen 2018 | 3 | 2 | 2 | 7 |
| Li 2016 | 4 | 2 | 3 | 9 |
| Li 2020 | 3 | 2 | 3 | 8 |
| Hamsten 1987 | 3 | 2 | 3 | 8 |
| Pavlov 2018 | 3 | 2 | 3 | 8 |
| Tofler 2016 | 4 | 2 | 3 | 9 |
| Giovannucci 2008 | 3 | 2 | 3 | 8 |
| Wang 2008 | 4 | 2 | 3 | 9 |
| Anderson 2010 | 4 | 2 | 3 | 9 |
| Brondum-Jacobsen 2012 | 4 | 2 | 3 | 9 |
| Peng, 2020 | 3 | 2 | 3 | 8 |
| Werner, 2014 | 3 | 2 | 3 | 8 |
| The maximum score possible for the involved criteria are: 4 in selection, 2 in comparability, and 3 in outcome. | | | | |

| **Supplementary Table 2. Newcastle-Ottawa quality assessment scale for case-control studies** | | | | |
| --- | --- | --- | --- | --- |
| **Study** | **Selection** | **Comparability** | **Outcome** | **Total score** |
| Morita 1993 | 2 | 2 | 3 | 7 |
| Hotta 2000 | 3 | 1 | 3 | 7 |
| Akbal, 2009 | 3 | 2 | 2 | 7 |
| Ramesh, 2020 | 3 | 2 | 2 | 7 |
| Elkhidir 2017 | 3 | 2 | 3 | 8 |
| Yang 2005 | 2 | 2 | 2 | 6 |
| Roy 2015 | 3 | 2 | 2 | 7 |
| The maximum point possible for the involved criteria are: 4 in selection, 2 in comparability, and 3 in outcome. | | | | |

| **Supplementary Table 3. Newcastle-Ottawa quality assessment scale adapted for cross-sectional studies** | | | | |
| --- | --- | --- | --- | --- |
| **Study** | **Selection** | **Comparability** | **Outcome** | **Total score** |
| Wang 2004 | 3 | 2 | 3 | 8 |
| Fonarow 2008 | 4 | 2 | 3 | 9 |
| Ng 2005 | 3 | 2 | 3 | 8 |
| Gardener 2011 | 3 | 2 | 3 | 8 |
| Shimabukuro 2003 | 2 | 2 | 3 | 7 |
| Xu 2006 | 3 | 2 | 3 | 8 |
| Bao 2011 | 3 | 2 | 3 | 8 |
| Hsu 2010 | 3 | 2 | 3 | 8 |
| Wang 2007 | 3 | 2 | 3 | 8 |
| Chesnaye 2016 | 3 | 2 | 3 | 8 |
| Zografos 2009 | 2 | 2 | 3 | 7 |
| Ni 2013 | 3 | 2 | 3 | 8 |
| Xiao 2013 | 3 | 2 | 3 | 8 |
| Choi 2008 | 3 | 2 | 3 | 8 |
| Barutcuoglu 2011 | 2 | 2 | 3 | 7 |
| Wang 2013 | 2 | 2 | 3 | 7 |
| Fang 2013 | 3 | 2 | 3 | 8 |
| Hao 2013 | 3 | 2 | 3 | 8 |
| Zhang 2008 | 3 | 2 | 3 | 8 |
| Lin 2010 | 2 | 2 | 3 | 7 |
| Bobbert 2013 | 3 | 2 | 3 | 8 |
| Chow 2013 | 3 | 2 | 3 | 8 |
| Bobbert 2010 | 3 | 2 | 3 | 8 |
| Liu 2014 | 3 | 2 | 3 | 8 |
| Majerczyk 2018 | 3 | 2 | 3 | 8 |
| Lambadiari 2014 | 3 | 2 | 3 | 8 |
| Sun 2013 | 3 | 2 | 3 | 8 |
| Vague 1986 | 2 | 2 | 3 | 7 |
| Wang 2017 | 3 | 2 | 3 | 8 |
| Verdoia 2014 | 3 | 2 | 3 | 8 |
| Tanik 2020 | 3 | 2 | 3 | 8 |
| Paquette, 2017 | 3 | 2 | 3 | 8 |
| Mba, 2019 | 4 | 2 | 3 | 9 |
| Toth, 2017 | 3 | 2 | 3 | 8 |
| Lee, 2013 | 2 | 2 | 3 | 7 |
| Bae, 2018 | 3 | 2 | 3 | 8 |
| The maximum point possible for the involved criteria are: 5 in selection, 2 in comparability, and 3 in outcome. | | | | |

| **Supplementary Table 4. AMSTAR-2 quality assessment of systematic reviews** | | | | | | | | | | | | | | | | | |
| --- | --- | --- | --- | --- | --- | --- | --- | --- | --- | --- | --- | --- | --- | --- | --- | --- | --- |
| **Study** | **Item 1** | **Item 2** | **Item 3** | **Item 4** | **Item 5** | **Item 6** | **Item 7** | **Item 8** | **Item 9** | **Item 10** | **Item 11** | **Item 12** | **Item 13** | **Item 14** | **Item 15** | **Item 16** | **Overall quality** |
| Liu 2018 | Y | PY | Y | PY | Y | Y | Y | PY | Y | N | Y | Y | Y | Y | Y | Y | High |
| Yang 2019 | Y | N | N | PY | Y | Y | PY | PY | Y | N | Y | Y | Y | Y | Y | Y | Low |
| Yarmolinsky 2016 | Y | Y | N | PY | Y | Y | PY | PY | Y | N | Y | N | Y | Y | Y | N | Moderate |
| Song 2017 | Y | N | Y | PY | Y | N | PY | PY | N | N | Y | N | N | N | N | Y | Critically low |
| Jung 2018 | Y | PY | N | PY | Y | Y | PY | PY | Y | N | Y | Y | Y | Y | Y | Y | Moderate |
| Abbreviations: N, no; PY, partial yes; Y, yes. | | | | | | | | | | | | | | | | | |
| The rating for quality is based on the evaluation of the seven critical domains (items 2, 4, 7, 9, 11, 13, 15): (i) “High”: no or one non-critical weakness; (ii) “Moderate”: more than one non-critical weakness; (iii) “Low”: one critical weakness with or without non-critical weaknesses; and (iv) “Critically low”: more than one critical weakness with or without non-critical weaknesses. | | | | | | | | | | | | | | | | | |

| **Supplementary Table 5. Summary of studies involving cardiac troponin I** | | | | | | |
| --- | --- | --- | --- | --- | --- | --- |
| **Author, year, country (reference)** | **Study design** | **Study population, n (mean age ± SD, years)** | **Male (%)** | **Specimen** | **Median/mean follow-up (years)** | **Findings** |
| You, 2007, Canada (41) | Cohort | ADHF patients, 2025 (76 ± 12) | 50 | NR | NR | Patients with cTnI levels >0.5 μg/L had a significantly higher risk of all-cause mortality compared with patients with lower cTnI levels. |
| Myhre, 2018, USA (42) | Cohort | HF patients with preserved ejection fraction, 236 (72) | 54 | Plasma | 2.6 | High hs-cTnI levels were associated with risk of cardiovascular death and HF hospitalization.  AUC: 0.75 for prediction of cardiovascular mortality. |
| Stelzle, 2017, UK (43) | Cohort | Patients with suspected ACS, 4748 (63 ± 16) | 57 | NR | 5 months | Patients with events had higher hs-cTnI levels than patients without events.  AUC increased from 0.82 to 0.87 when hs-cTnI was added to the traditional risk factors model for prediction of HF hospitalization and cardiac death. |
| Cavender, 2017, USA (44) | Cohort | T2DM patients with stabilized ACS, 3808 (60) | 67 | Serum | 1.5 | Patients with hs-cTnI levels ≥99th percentile were at increased risk of cardiovascular death, myocardial infarction, or stroke compared with patients with lower hs-cTnI levels. |
| White, 2014, Australia (45) | Cohort | Stable CAD patients, 7863 (62) | 83 | Plasma | 6 | Higher cTnI levels were predictive of subsequent myocardial infarction and cardiovascular mortality.  AUC: 0.67. NRI: 4.8%. |
| Tahhan, 2018, USA (46) | Cohort | Patients undergoing heart catheterization, 3087 (63 ± 12) | 64 | Plasma | 5.2 | Higher hs-cTnI levels in patients with more severe CAD than patients with normal coronary angiography.  High hs-cTnI levels were associated with risk of cardiovascular mortality or myocardial infarction.  Addition of hs-cTnI to model with traditional risk factors improved the AUC from 0.68 to 0.71 for prediction of adverse cardiovascular events. |
| Abbreviations: ACS, acute coronary syndrome; ADHF, acute decompensated heart failure; AUC, area under curve; CAD, coronary artery disease; HF, heart failure; hs-cTnI, high-sensitivity cardiac troponin I; NR, not reported; NRI, net reclassification index; T2DM, type 2 diabetes mellitus. | | | | | | |

| **Supplementary Table 6. Summary of studies involving BNP/NT-proBNP** | | | | | | |
| --- | --- | --- | --- | --- | --- | --- |
| **Author, year, country (reference)** | **Study design** | **Study population, n (mean age ± SD, years)** | **Male (%)** | **Specimen** | **Median/mean follow-up (years)** | **Findings** |
| Masson, 2008, Italy (48) | Cohort | Patients with stable symptomatic HF, 1742 (63 ± 11) | 81 | NR | 2 | High NT-proBNP levels at baseline and 4 months were associated with a significant risk of all-cause mortality during a median follow-up of 2 years.  AUC=0.66 (NT-proBNP at baseline); AUC=0.70 (NT-proBNP at 4 months) for prediction of all-cause mortality. |
| Wang, 2004, USA (49) | Cross-sectional | Individuals without HF from Framingham Heart Offspring Study, 3532 (59) | 47 | Plasma | N/A | Lower plasma BNP in obese individuals than in lean individuals.  BNP levels were inversely correlated with body mass index. |
| Morita, 1993, Japan (50) | Case-control | Acute MI patients, 50 (66 ± 2) | 72 | Plasma | N/A | Higher plasma BNP levels in patients than in controls.  Plasma BNP increased markedly in the early phase of acute MI. |
| Omland, 2007, Norway (51) | Cohort | Patients with stable CAD and preserved LV function, 3761 (64) | 81 | Plasma | 4.8 | High plasma BNP levels were associated with risk of congestive heart failure during a median follow-up of 4.8 years.  AUC increased from 0.82 to 0.84 when BNP was added to the conventional risk factor model. |
| Bhalla, 2004, USA (52) | Cohort | T2DM patients, 482 (57) | 95 | Plasma | 2.3 | Patients with BNP levels ≥120 pg/mL had a higher risk of all-cause mortality than patients with lower BNP levels.  AUC: 0.72. |
| Paget, 2011, France (53) | Cohort | Hypertensive patients, 684 (52) | 53 | Plasma | 5.7 | High NT-proBNP levels were associated with all-cause mortality in hypertensive patients and in patients without LV hypertrophy. |
| Wolsk, 2017, Denmark (54) | Cohort | Patients with ACS and T2DM, 5525 (62) | 70 | NR | 2.2 | Higher BNP or NT-proBNP levels were associated with adverse cardiovascular outcomes.  Addition of BNP or NT-proBNP to risk factor model significantly improved the AUC from 0.77 to 0.83 for prediction of cardiovascular death.  BNP: NRI=36.0%; IDI=5.6%. NT-proBNP: NRI=30.9%; IDI=4.0%. |
| Fonarow, 2008, USA (55) | Cross-sectional | Patients hospitalized with HF, 42636 patient episodes (74 ± 11) | 49 | NR | N/A | Patients with BNP ≥840 pg/mL and increased cardiac troponin levels were at increased mortality risk compared with patients with lower BNP and no increase in cardiac troponin. |
| Mishra, 2014, USA (56) | Cohort | CAD patients, 983 (67) | 81 | Plasma | 6.5 | High BNP levels were associated with increased risk of MACE.  Addition of BNP to risk factor model significantly increased the AUC from 0.63 to 0.72 for prediction of MACE.  NRI: 56%. IDI: 11%. |
| Hillis, 2014, Australia (57) | Cohort | T2DM patients, 3862 (67 ± 7) | 61 | Plasma | 5 | High NT-proBNP and hs-cTnT levels were strongly associated with cardiovascular events and mortality.  Addition of NT-proBNP to risk factor model significantly improved the AUC from 0.70 to 0.74, and the combination of NT-proBNP and hs-cTnT improved the AUC further to 0.75 for prediction of cardiovascular events. |
| Abbreviations: ACS, acute coronary syndrome; AUC, area under curve; BNP, B-type natriuretic peptide; CAD, coronary artery disease; hs-cTnT, high-sensitivity cardiac troponin T; HF, heart failure; IDI, integrated discrimination improvement; LV, left ventricular; MACE, major adverse cardiovascular event; MI, myocardial infarction; N/A, not applicable; NT-proBNP, N-terminal pro-B-type natriuretic peptide; NR, not reported; NRI, net reclassification index; T2DM, type 2 diabetes mellitus. | | | | | | |

| **Supplementary Table 7. Summary of studies involving adiponectin** | | | | | | |
| --- | --- | --- | --- | --- | --- | --- |
| **Author, year, country (reference)** | **Study design** | **Study population, n (mean age ± SD, years)** | **Male (%)** | **Specimen** | **Median/mean follow-up (years)** | **Findings** |
| Hotta, 2000, Japan (58) | Case-control | T2DM patients = 183 (60)  Healthy controls = 82 (57) | 69; 66 | Plasma | N/A | Lower plasma adiponectin in patients than in controls.  Adiponectin levels were negatively correlated with body mass index, fasting plasma glucose and insulin levels. |
| Ng, 2005, Australia (59) | Cross-sectional | Healthy males, 41 (47 ± 9) | 100 | Plasma | N/A | Plasma adiponectin was inversely associated with triglycerides, cholesterol and VLDL apolipoprotein B concentrations. |
| Inoue, 2007, Japan (60) | Cohort | CAD patients, 149 (63 ± 9) | 71 | Plasma | 7 | Lower adiponectin levels in patients with multivessel CAD than patients with single vessel CAD.  Low adiponectin was associated with cardiovascular events during 7 years of follow-up. |
| Gardener, 2011, USA (61) | Cross-sectional | Healthy individuals, 1522 (66 ± 9) | 40 | Plasma | N/A | Low plasma adiponectin was associated with increased carotid IMT. |
| Shimabukuro, 2003, Japan (62) | Cross-sectional | Healthy individuals, 76 | 100 | Plasma | N/A | Low plasma adiponectin levels were closely correlated with resistance vessel endothelial dysfunction. |
| Liu, 2018, China (63) | Meta-analysis | 7 studies published between 2005 and 2015 for accuracy analysis  Inclusion criteria: studies involved adiponectin measurement upon diagnosis of metabolic syndrome  Sample size = 1248 participants | N/A | N/A | N/A | AUC for accuracy of adiponectin upon metabolic syndrome diagnosis ranging from 0.67 to 0.89. |
| Yang, 2019, China (64) | Meta-analysis | 12 prospective studies published between 2006 and 2016  Inclusion criteria: studies involved CAD patients and adiponectin measurement  Total sample size = 10974 CAD patients | N/A | N/A | N/A | High adiponectin levels were associated with cardiovascular and all-cause mortality. |
| Oliveira, 2013, Brazil (65) | Cohort | Patients with ACS, 114 (62 ± 11) | 59 | Serum | 1.1 | Adiponectin was associated with higher risk of cardiovascular events. Significant correlation between adiponectin and BNP. |
| Pratesi, 2016, Italy (66) | Cohort | Stable CAD patients, 138 (69 ± 10) | 88 | Plasma | 3.8 | Patients with adiponectin levels >13.2 ng/mL had a significantly higher risk of all-cause mortality than patients with lower adiponectin levels.  AUC: 0.78. |
| Abbreviations: ACS, acute coronary syndrome; AUC, area under curve; BNP, B-type natriuretic peptide; CAD, coronary artery disease; IMT, intima-media thickness; N/A, not applicable; T2DM, type 2 diabetes mellitus; VLDL, very low-density lipoprotein. | | | | | | |

| **Supplementary Table 8. Summary of studies involving A-FABP** | | | | | | |
| --- | --- | --- | --- | --- | --- | --- |
| **Author, year, country (reference)** | **Study design** | **Study population, n (mean age ± SD, years)** | **Male (%)** | **Specimen** | **Median/mean follow-up (years)** | **Findings** |
| Xu, 2006, China (67) | Cross-sectional | Overweight/obese individuals = 129 (54)  Lean individuals = 100 (58) | 67; 54 | Serum | N/A | Higher serum A-FABP in overweight/obese than in lean individuals.  Serum A-FABP was positively correlated with waist circumference, blood pressure, dyslipidemia, fasting insulin and the HOMA-IR index. |
| Bao, 2011, China (68) | Cross-sectional | Adults undergoing coronary angiography, 341 (65) | 65 | Serum | N/A | Higher serum A-FABP in patients with CAD than non-CAD subjects.  Significant positive correlation between serum A-FABP and the severity of coronary atherosclerosis. |
| Hsu, 2010, Taiwan (69) | Cross-sectional | CAD patients, 98 (68 ± 11) | 75 | Serum | N/A | A-FABP was positively correlated with metabolic syndrome.  Significant correlation between serum A-FABP levels and number of metabolic syndrome diagnostic criteria. |
| Huang, 2018, Taiwan (70) | Cohort | CAD patients, 106 (66 ± 9) | 77 | Serum | 4.4 | High serum A-FABP levels were associated with adverse cardiovascular events. |
| Takagi, 2017, Japan (71) | Cohort | Patients with stable angina undergoing PCI, 130 (72 ± 9) | 81 | Serum | 4.2 | Serum A-FABP was an independent predictor of long-term cardiovascular events. |
| Eynatten, 2012, Germany (72) | Cohort | CAD patients, 1069 (60) | 86 | Serum | 10 | High A-FABP levels were associated with cardiovascular mortality during 10-years follow-up. |
| Chow, 2013, China (73) | Cohort | General population, 849 men and 998 women (mean: 57) | 54 | Serum | 9.4 | High A-FABP levels were associated with long-term adverse cardiovascular events.  NRI and IDI significantly improved by the addition of A-FABP to the traditional risk factor model (NRI: 18.6%; IDI: 0.25%). |
| Reiser, 2015, Switzerland (74) | Cohort | STEMI = 269 (63)  NSTEMI = 170 64)  Stable CAD = 68 (62)  Controls = 313 (60) | 77; 81; 87; 68 | Serum | 30-day | Higher serum A-FABP levels in STEMI patients than controls.  High serum A-FABP levels in patients with ACS were associated with cardiovascular events during 30-day follow-up.  AUC = 0.65 for A-FABP alone; AUC = 0.68 for the combination of A-FABP and NT-proBNP. |
| Abbreviations: ACS, acute coronary syndrome; A-FABP, adipocyte fatty acid-binding protein; AUC, area under curve; NT-proBNP, N-terminal pro-B-type natriuretic peptide; CAD, coronary artery disease; HOMA-IR, homeostatic model assessment of insulin resistance; IDI, integrated discrimination improvement; N/A, not applicable; NRI, net reclassification index; NSTEMI, non ST-elevation myocardial infarction; PCI, percutaneous coronary intervention; STEMI, ST-elevation myocardial infarction. | | | | | | |

| **Supplementary Table 9. Summary of studies involving H-FABP** | | | | | | |  |
| --- | --- | --- | --- | --- | --- | --- | --- |
| **Author, year, country (reference)** | **Study design** | **Study population, n (mean age ± SD, years)** | **Male (%)** | **Specimen** | **Median/**  **mean follow-up (years)** | **Findings** | |
| Akbal, 2009, Turkey (75) | Case-control | Patients with metabolic syndrome = 55 (55) Healthy controls = 73 (50) | 40; 44 | Serum | N/A | Higher serum H-FABP levels in patients with metabolic syndrome than control subjects. | |
| Ramesh, 2020, India (76) | Case-control | Prediabetics = 50  Healthy controls = 50 (mean: 36) | 48; 54 | Serum | N/A | Higher serum H-FABP levels in prediabetic patients than in controls. Serum H-FABP levels were positively correlated with carotid IMT and high-sensitivity CRP. | |
| O'Donoghue, 2016, USA (77) | Cohort | Patients with ACS, 2287 | 72 | Serum | 10-month | High H-FABP levels were associated with the composite endpoint of all-cause mortality, non-fatal MI, or CHF. | |
| Niizeki, 2005, Japan (78) | Cohort | Patients with CHF, 186 (67 ± 12) | 59 | Serum | 1.5 | Patients with H-FABP levels >4.3 ng/ml had a higher risk of cardiac events compared with patients with lower H-FABP levels. AUC: 0.79. | |
| Zhang, 2020, China (79) | Cohort | Stable CAD patients, 4594 (58 ± 10) | 72 | Plasma | 7.1 | High H-FABP levels were associated with adverse cardiovascular events in CAD patients with pre-DM and DM. | |
| Abbreviations: ACS, acute coronary syndrome; AUC, area under curve; CAD, coronary artery disease; CHF, chronic heart failure; DM, diabetes mellitus; HbA1c, hemoglobin A1c; H-FABP, heart-type fatty acid-binding protein; IFG, impaired fasting glucose; IGT, impaired glucose tolerance; IMT, intima-media thickness; MI, myocardial infarction; N/A, not applicable; NGT, normal glucose tolerance. | | | | | | |  |

| **Supplementary Table 10. Summary of studies involving lipocalin-2** | | | | | | |
| --- | --- | --- | --- | --- | --- | --- |
| **Author, year, country (reference)** | **Study design** | **Study population, n (mean age ± SD, years)** | **Male (%)** | **Specimen** | **Median/**  **mean follow-up (years)** | **Findings** |
| Wang, 2007, China (18) | Cross-sectional | Overweight individuals = 80  Obese individuals = 49  Lean individuals = 100  (33-72) | NR | Serum | N/A | Serum lipocalin-2 was positively correlated with waist circumference, blood pressure, hyperglycemia, hypertriglyceridemia and the HOMA-IR index.  Significant correlation between lipocalin-2 and high-sensitivity CRP. |
| Chesnaye, 2016, Mexico (82) | Cross-sectional | Healthy individuals, 24 men and 29 women (mean: 41 ± 10) | 45 | Serum | N/A | Significantly higher lipocalin-2 levels in males than females.  Plasma lipocalin-2 levels were correlated with age, body mass index, HOMA-IR, triglycerides, and high-density lipoprotein. |
| Elkhidir, 2017, Sudan (83) | Case-control | T2DM patients = 57 (51)  Non-diabetic controls = 30 (50) | 51 | Serum | N/A | Higher serum lipocalin-2 in T2DM patients than controls. |
| Zografos, 2009, Greece (84) | Cross-sectional | Angiographically confirmed CAD = 31 (65)  Controls with normal coronary arteries = 42 (58) | 87; 67 | Serum | N/A | Higher serum lipocalin-2 levels in patients with CAD than controls with normal coronary arteries. Serum lipocalin-2 was correlated with the severity of CAD. |
| Ni, 2013, China (85) | Cross-sectional | Angiographically confirmed CAD = 188 (67)  Controls with normal coronary arteries = 73 (64) | 65 | Serum | N/A | Higher lipocalin-2 levels in patients with CAD than non-CAD subjects.  Serum lipocalin-2 was positively correlated with the number of metabolic syndrome components in men. |
| Xiao, 2013, China (86) | Cross-sectional | T2DM patients divided into two groups  Patients with subclinical atherosclerosis = 78 (57)  Patients without atherosclerosis = 206 (53) | 49; 51 | Serum | N/A | Significant difference in serum lipocalin-2 levels between patients with and without subclinical atherosclerosis.  Positive correlation between lipocalin-2 and carotid IMT. |
| Wu, 2014, China (87) | Cohort | General population  287 men and 420 women (mean: 59 ± 14) | 41 | Serum | 6.2 | High lipocalin-2 levels were associated with cardiovascular events in male subjects.  Addition of lipocalin-2 to traditional risk factor model improved the AUC from 0.77 to 0.81. |
| Choi, 2008, Korea (88) | Cross-sectional | 49 Stable CAD patients and 42 controls  (mean: 61) | 60 | Serum | N/A | Significantly higher lipcoalin-2 levels in CAD patients than healthy controls. |
| Yndestad, 2009, Norway (89) | Cohort | Patients with CHF, 150 (56 ± 12) | 87 | Serum | 2.3 | Patients in NYHA class III/IV had higher lipocalin-2 levels than controls.  High lipocalin-2 levels were associated with the composite endpoint of non-fatal MI, stroke, and cardiovascular and total mortality. |
| Akcay, 2012, Turkey (90) | Cohort | PCI treated STEMI patients, 106 (52) | 74 | Serum | 1 | Patients with lipocalin-2 levels >46 ng/mL had higher incidence of MACE and death compared with patients with lower lipocalin-2 levels.  AUC = 0.76 for prediction of mortality. |
| Avci, 2020, Turkey (91) | Cohort | STEMI patients divided into two groups  Preserved LVEF = 34 (56)  Reduced LVEF = 34 (67) | 82 | Serum | 6-month | High lipocalin-2 levels were associated with cardiovascular mortality in STEMI patients.  AUC: 0.85 |
| Bolignano, 2009, Italy (92) | Cohort | Elderly with CHF, 46 (78 ± 7) | 65 | Serum | 1.6 | Patients with lipocalin-2 levels >783 ng/mL had a significantly higher risk of mortality compared to patients with lower lipocalin-2 levels.  AUC: 0.68. |
| Abbreviations: AUC, area under curve; CAD, coronary artery disease; CHF, chronic heart failure; CRP, c-reactive protein; HOMA-IR, homeostatic model assessment of insulin resistance; IMT, intima-media thickness; LVEF, left ventricular ejection fraction; MACE, major adverse cardiovascular event; MI, myocardial infarction; N/A, not applicable; NR, not reported; NYHA, New York Heart Association; PCI, percutaneous coronary intervention; STEMI, ST-elevation myocardial infarction; T2DM, type 2 diabetes mellitus. | | | | | | |

| **Supplementary Table 11. Summary of studies involving FGF-19 and FGF-21** | | | | | | |
| --- | --- | --- | --- | --- | --- | --- |
| **Author, year, country (reference)** | **Study design** | **Study population, n (mean age ± SD, years)** | **Male (%)** | **Specimen** | **Median/**  **mean follow-up (years)** | **Findings** |
| Barutcuoglu, 2011, Turkey (20) | Cross-sectional | T2DM with metabolic syndrome = 26 (54)  Healthy controls = 12 (50) | 46 | Serum | N/A | Lower serum FGF-19 levels in patients with metabolic syndrome than controls.  FGF-19 levels were negatively correlated with body mass index and HbA1c. |
| Wang, 2013, China (21) | Cross-sectional | 30 GDM patients and 60 controls (29 ± 3) | NR | Serum | N/A | Serum FGF-19 levels were significantly lower in patients than in controls, in contrast with FGF-21 levels. Both serum FGF-19 and FGF-21 levels were strongly correlated with HOMA-IR and adiponectin. |
| Fang, 2013, China (94) | Cross-sectional | T2DM patients = 104 (50)  IFG = 91 (50)  IGT = 93 (48)  NGT = 81 (41) | 55; 47; 46; 57 | Serum | N/A | Lower serum FGF-19 levels in patients with IFG and T2DM than in NGT subjects.  Serum FGF-19 levels were inversely associated with fasting glucose levels. |
| Hao, 2013, China (95) | Cross-sectional | Angiographically confirmed CAD = 228 (67)  Controls with normal coronary arteries = 87 (65) | 71; 51 | Serum | N/A | Lower serum FGF-19 levels in CAD patients than non-CAD subjects.  Low serum FGF-19 was associated with the presence and severity of CAD. |
| Zhang, 2008, China (96) | Cross-sectional | Overweight/obese individuals = 127 (55)  Lean individuals = 105 (57) | 49; 50 | Serum | N/A | Serum FGF-21 was positively correlated with body mass index, fasting insulin, triglycerides, HOMA-IR and serum A-FABP levels. |
| Lin, 2010, China (97) | Cross-sectional | 135 CAD patients and 61 controls (69 ± 6) | 51 | Serum | N/A | Higher serum FGF-21 levels in patients than controls.  High serum FGF-21 was associated with fasting glucose, apolipoprotein A1 and triglyceride. |
| Bobbert, 2013, Germany (98) | Cross-sectional | Healthy individuals, 440 | 34 | Plasma | N/A | FGF-21 was associated with incident metabolic syndrome, future T2DM and progression to impaired glucose metabolism. |
| Chen, 2011, China (99) | Cohort | T2DM = 298 (59)  IFG/IGT = 558 (55)  NGT = 1044 (49) | 50; 51; 43 | Plasma | 5.4 | Higher plasma FGF-21 levels in IGT/IFG vs. T2DM and NGT.  High FGF-21 levels predicted the development of T2DM. |
| Chow, 2013, China (100) | Cross-sectional | Adults undergoing carotid IMT measurement, 285 men and 385 women (mean: 58) | 43 | Serum | N/A | High serum FGF-21 was associated with carotid IMT in women. |
| Chen, 2018, China (101) | Cohort | Patients with acute MI, 165 (64 ± 11) | 85 | Serum | 2 | Patients with FGF-21 levels ≥123 pg/mL had a higher risk of MACE compared with patients with lower FGF-21 levels.  AUC: 0.67. |
| Shen, 2018, China (102) | Cohort | CAD patients, 169 (67) | 69 | Serum | 2 | High serum FGF-21 was associated with MACE in CAD patients.  AUC: 0.67. |
| Li, 2016, China (103) | Cohort | CAD patients, 1668 (63) | 66 | Serum | 4.9 | High serum FGF-21 levels were associated with cardiovascular and all-cause mortality during a median follow-up of 4.9 years. |
| Abbreviations: A-FABP, adipocyte fatty acid-binding protein; AUC, area under curve; CAD, coronary artery disease; FGF, fibroblast growth factor; GDM, gestational diabetes mellitus; HbA1c, hemoglobin A1c; HOMA-IR, homeostatic model assessment of insulin resistance; IFG, impaired fasting glucose; IGT, impaired glucose tolerance; IMT, intima-media thickness; MACE, major adverse cardiovascular event; MI, myocardial infarction; N/A, not applicable; NGT, normal glucose tolerance; NR, not reported; T2DM, type 2 diabetes mellitus. | | | | | | |

| **Supplementary Table 12. Summary of studies involving RBP4** | | | | | | |
| --- | --- | --- | --- | --- | --- | --- |
| **Author, year, country (reference)** | **Study design** | **Study population, n (mean age, years)** | **Male (%)** | **Specimen** | **Median/**  **mean follow-up (years)** | **Findings** |
| Yang, 2005, USA (105) | Case-control | Obese diabetic = 10 (58)  Obese non-diabetic = 7 (48)  Lean non-diabetic = 5 (38) | NR | Serum | N/A | Serum RBP4 levels were increased in obesity and T2DM. |
| Bobbert, 2010, Germany (22) | Cross-sectional | Adults undergoing carotid IMT measurement, 96 (55) | 46 | Plasma | N/A | Significant correlation between RBP4 and carotid IMT. |
| Liu, 2014, China (23) | Cross-sectional | Healthy individuals, 1748 (65) | 52 | Plasma | N/A | High plasma RBP4 levels were associated with metabolic syndrome and its components including hypertriglyceridemia, reduced HDL cholesterol and hyperglycemia. RBP4 was strongly correlated with inflammatory markers and oxidative stress. |
| Majerczyk, 2018, Poland (106) | Cross-sectional | Elderly, 3038 (78) | 52 | Plasma | N/A | High serum RBP4 levels were associated with the presence and the number of components of metabolic syndrome. |
| Lambadiari, 2014, Greece (107) | Cross-sectional | Angiographically confirmed CAD = 305 (65)  Controls with normal coronary arteries = 91 (62) | 88; 81 | Serum | N/A | Higher serum RBP4 in CAD patients than non-CAD subjects. AUC= 0.72 for the discrimination between presence and absence of CAD.  RBP4 was positively associated with CAD severity in patients with CAD. |
| Sun, 2013, USA (108) | Cross-sectional | 468 female patients with CAD and 472 controls  (59) | NR | Plasma | N/A | High RBP4 levels were associated with increased risk of CAD during the first 8 years of follow-up. |
| Li, 2020, China (109) | Cohort | Elderly with CHF, 934 (69) | 65 | Serum | 2 | High serum RBP4 was associated with the composite endpoint of cardiovascular mortality and CHF rehospitalization.  AUC: 0.74. |
| Abbreviations: AUC, area under curve; CAD, coronary artery disease; CHF, chronic heart failure; HDL, high-density lipoprotein; IMT, intima-media thickness; N/A, not applicable; NR, not reported; RBP, retinol-binding protein; T2DM, type 2 diabetes mellitus. | | | | | | |

| **Supplementary Table 13. Summary of studies involving PAI-1** | | | | | | |
| --- | --- | --- | --- | --- | --- | --- |
| **Author, year, country (reference)** | **Study design** | **Study population, n (mean age ± SD, years)** | **Male (%)** | **Specimen** | **Median/**  **mean follow-up (years)** | **Findings** |
| Vague, 1986, Belgium (113) | Cross-sectional | Healthy individuals, 35 (15-45) | NR | NR | N/A | PAI-1 was positively correlated with body mass index and plasma insulin levels. |
| Yarmolinsky, 2016, Brazil (114) | Meta-analysis | 52 epidemiological studies published between 1988 and 2012  Inclusion criteria: studies involved assessment of T2DM and PAI-1 measurement  Total sample size = 17209 participants | N/A | N/A | N/A | High PAI-1 levels were associated with the risk of T2DM. |
| Hamsten, 1987, Sweden (115) | Cohort | Male patients with a first MI, 109 (40 ± 3) | NR | Plasma | 3 | High PAI-1 levels were independently associated with reinfarction. |
| Song, 2017, USA (116) | Meta-analysis | 14 studies published between 1992 and 2016  Inclusion criteria: studies involved CAD and PAI-1 measurement | N/A | N/A | N/A | High PAI-1 levels were associated with the risk of CAD. |
| Jung, 2018, Canada (117) | Meta-analysis | 38 studies published between 1991 and 2016  Inclusion criteria: studies involved components of MACE and PAI-1 measurement  Total sample size = 11557 participants | N/A | N/A | N/A | High PAI-1 levels were associated with MACE in both primary and secondary event populations. |
| Pavlov, 2018, Croatia (118) | Cohort | STEMI patients treated with primary PCI, 87 (61 ± 12) | 71 | NR | 5 | Patients with PAI-1 activity rise >3.7 U/mL had a higher risk of 5-year mortality.  AUC: 0.75. |
| Tofler, 2016, USA (119) | Cohort | Individuals without prior CVD from Framingham Heart Offspring Study, 3203 (55) | 45 | Antigen | 6 | Increasing PAI-1 levels were independently associated with subsequent major cardiovascular events.  Addition of PAI-1 to traditional risk factor model modestly improved the AUC from 0.808 to 0.812. NRI: 4.8%. |
| Abbreviations: AUC, area under curve; CAD, coronary artery disease; CVD, cardiovascular disease; MACE, major adverse cardiovascular event; MI, myocardial infarction; N/A, not applicable; NR, not reported; NRI, net reclassification index; PAI, plasminogen activator inhibitor; PCI, percutaneous coronary intervention; STEMI, ST-elevation myocardial infarction; T2DM, type 2 diabetes mellitus. | | | | | | |

| **Supplementary Table 14. Summary of studies involving 25-hydroxyvitamin D** | | | | | | |
| --- | --- | --- | --- | --- | --- | --- |
| **Author, year, country (reference)** | **Study design** | **Study population, n (mean age ± SD, years)** | **Male (%)** | **Specimen** | **Median/mean follow-up (years)** | **Findings** |
| Wang, 2017, China (121) | Cross-sectional | T2DM patients undergoing carotid IMT measurement, 314 (52) | 62 | Serum | N/A | Low serum 25-OH D3 levels were associated with the presence of carotid atherosclerotic plaque. |
| Verdoia, 2014, Italy (122) | Cross-sectional | Adults undergoing coronary angiography, 1484 (68) | 71 | Serum | N/A | Significantly lower 25-OH D3 in patients with significant CAD than those without coronary disease.  Low 25-OH D3 levels were associated with a higher prevalence of severe CAD. |
| Roy, 2015, India (123) | Case-control | 120 acute MI patients and 120 controls  (52) | 88; 88 | Serum | N/A | Lower serum 25-OH D levels in acute MI patients than controls.  Vitamin D deficiency (25-OH D <10 ng/mL) was associated with a higher risk of acute MI. |
| Giovannucci, 2008, USA(124) | Cohort | 454 male patients with MI and 900 controls (64) | NR | Plasma | 10 | Patients with deficient levels of 25-OH D (≤15 ng/mL) had a higher risk of MI compared with patients with sufficient 25-OH D levels (≥30 ng/mL). |
| Wang, 2008, USA (125) | Cohort | Individuals without prior CVD from Framingham Heart Offspring Study, 1739 (59 ± 9) | 45 | Serum | 5.4 | Vitamin D deficiency (25-OH D <15 ng/mL) was associated with the risk of cardiovascular events. |
| Anderson, 2010, USA (126) | Cohort | General population, 41504 (55 ± 21) | 25 | Serum | 1.3 | Patients with vitamin D deficiency (25-OH D <15 ng/mL) had a higher prevalence of cardiovascular risk factors.  Vitamin D deficiency was associated with adverse cardiovascular events in patients aged 50 years and older. |
| Brondum-Jacobsen, 2012, Denmark (127) | Cohort | General population, 10170 (57) | 44 | Plasma | 21 | Decreasing plasma 25-OH D levels were associated with increased risk of ischemic heart disease, MI, and early death. |
| Tanik, 2020, Turkey (128) | Cross-sectional | Patients with APE, 99 (66 ± 17) | 37 | Serum | N/A | Low serum 25-OH D levels were associated with in-hospital mortality.  25-OH D cutoff to predict in-hospital mortality ≤6.47 ng/mL. AUC: 0.81. |
| Abbreviations: 25-OH, 25-hydroxyvitamin; APE, acute pulmonary embolism; AUC, area under curve; CAD, coronary artery disease; CVD, cardiovascular disease; IMT, intima-media thickness; MI, myocardial infarction; N/A, not applicable; NR, not reported; T2DM, type 2 diabetes mellitus. | | | | | | |

| **Supplementary Table 15. Summary of studies involving PCSK9** | | | | | | |
| --- | --- | --- | --- | --- | --- | --- |
| **Author, year, country (reference)** | **Study design** | **Study population, n (mean age ± SD, years)** | **Male (%)** | **Specimen** | **Median/**  **mean follow-up (years)** | **Findings** |
| Paquette, 2017, Kenya (129) | Cross-sectional | Patients with metabolic syndrome = 141 (45) Healthy controls = 1179 (37) | 31; 43 | Plasma | N/A | Higher plasma PCSK9 in patients with metabolic syndrome than controls. Plasma PCSK9 was positively correlated with lipid parameters. |
| Mba, 2019, Cameroon (130) | Cross-sectional | T2DM = 54 Obese = 54 Lean = 54 (mean: 55) | 45 | Plasma | N/A | Higher PCSK9 levels in obese and T2DM individuals than in lean controls. Plasma PCSK9 was positively correlated with BMI. |
| Toth, 2017, Slovakia (131) | Cross-sectional | Obese = 40 (37) Overweight = 30 (37) Normal weight = 50 (32) | 66; 54 | Plasma | N/A | Higher plasma levels of PCSK9 in obese patients than non-obese. Significant correlation between PCSK9 levels and subclinical vascular changes. |
| Lee, 2013, Korea (132) | Cross-sectional | Hypertensive patients, 126 (63 ± 10) | 55 | Serum | N/A | Serum PCSK9 was associated with carotid IMT. |
| Bae, 2018, Korea (133) | Cross-sectional | Patients with suspected ACS, 121 (59) | NR | Serum | N/A | Higher serum PCSK9 in patients with coronary artery lesions. Significant positive association between PCSK9 and the severity of coronary artery occlusion. |
| Peng, 2020, China (134) | Cohort | Stable CAD patients, 1225 (58 ± 10) | 68 | Plasma | 3.3 | Plasma PCSK9 was positively correlated with lipid profiles, fasting glucose and HbA1c. High PCSK9 levels were associated with MACE in stable CAD patients with T2DM. |
| Werner, 2014, Germany (135) | Cohort | Stable CAD patients, 504 (68) | 83 | Serum | 4 | High PCSK9 concentrations were associated with cardiovascular events in patients with stable CAD on statin treatment. |
| Abbreviations: ACS, acute coronary syndrome; CAD, coronary artery disease; HbA1c, hemoglobin A1c; IMT, intima-media thickness; MACE, major adverse cardiovascular event; N/A, not applicable; NR, not reported; PCSK9, proprotein convertase subtilisin/kexin type 9; T2DM, type 2 diabetes mellitus. | | | | | | |
